# Perceptions of newsworthiness are contaminated by a political usefulness bias

**DOI:** 10.1098/rsos.172239

**Published:** 2018-08-01

**Authors:** Harold Pashler, Gail Heriot

**Affiliations:** 1 Department of Psychology, University of California, San Diego, 9500 Gilman Drive 0109, La Jolla, CA 92093-0109, USA; 2 School of Law, University of San Diego, San Diego, CA 92110, USA

**Keywords:** judgement, motivated reasoning, political psychology

## Abstract

Are people's perceptions of the newsworthiness of events biased by a tendency to rate as more important any news story that seems likely to lead others to share their own political attitudes? To assess this, we created six pairs of hypothetical news stories, each describing an event that seemed likely to encourage people to adopt attitudes on the opposite side of a particular controversial issue (e.g. affirmative action and gay marriage). In total, 569 subjects were asked to evaluate the importance of these stories ‘to the readership of a general-circulation newspaper’, disregarding how interesting they happened to find the event. Subjects later indicated their own personal attitudes to the underlying political issues. Predicted crossover interactions were confirmed for all six issues. All the interactions took the form of subjects rating stories offering ‘ammunition’ for their own side of the controversial issue as possessing greater intrinsic news importance.

## 1. Introduction

Editors and managers of media outlets (whether these outlets are newspapers, television, magazines or news websites) must constantly assess the importance and newsworthiness of different potential news stories, which compete for time and attention. The nature of these judgements has been the focus of only a small amount of empirical research. The current study examined an unselected sample of US adults, instructing them to rate the importance of hypothetical news stories as if they were the editor of a city newspaper. The results showed that their newsworthiness judgements were contaminated by a strong tendency to rate as more important events that seemed likely to provide useful ‘political ammunition’ for the raters' own political opinions.

### 1.1. Prior research on newsworthiness

The topic of newsworthiness assessment has received less study than one might have expected given its importance. A very influential paper by Galtung & Ruge [1] was perhaps the first to offer an explicit set of principles for what draws news attention, suggesting, for example, an emphasis on negative events among many other principles (see [2] for a discussion and effort to update). A systematic and thoughtful review by Shoemaker & Cohen [3] presented a broad theory of newsworthiness bolstered by interviews with journalists from 10 countries. According to Shoemaker and Cohen's theory, two distinct factors underlie perceptions of newsworthiness: *deviancy* and *social significance.* Deviancy refers to unusualness. Thus, the authors point out, the 9/11 attacks on the USA score high in both deviancy and social significance, while the death of the UK's Princess Diana scores high only on deviancy. While the authors did not attempt a precise definition of social significance, they made it clear that the key factor was whether the news development had implications for large-scale future developments within a given society. The authors make a rather compelling case that both deviancy and social significance play an independent role in newsworthiness judgements. They do not, however, discuss the possibility that newsworthiness judgements are subject to any intrinsic partisan or political biases, and indeed the index of their book does not include an entry for ‘bias’ or other similar concepts.

### 1.2. Current approach

In the current study, we asked whether people conflate newsworthiness of hypothetical news stories with the likelihood that these stories would provide useful ‘political ammunition’ for those sharing their own policy preferences. Subjects were instructed to rate the newsworthiness of 12 hypothetical news stories (along with eight filler stories). The 12 stories consisted of six pairs of stories bearing (sometimes directly, sometimes less directly) on a particular highly controversial topic. The topics were government aid for the poor, affirmative action, defence spending, police racial bias, gay marriage and environmental conservation. Pairs were constructed so that one item within the pair would tend to provide useful ‘political ammunition’ for one side of the relevant issue, and the other item provided ammunition on the opposite side. This classification was made by examining the arguments often made by debaters advocating each side of the relevant issue.

For example, on the issue of whether government aid for the poor is too small or too great in the USA, the two paired hypothetical news stories were

1. A welfare recipient in your city was arrested for fraudulently receiving $25 000 worth of welfare cheques over a 2 year period.
2. A new study shows that food stamp recipients in your state do not receive enough food stamps to cover the cost of preparing nutritious meals for their households (as defined by the US Government Department of Health and Human Services).

Item 1 resembles the points frequently made by conservative politicians advocating limitations in welfare benefits going back as far as Reagan [4] and probably much further, whereas item 2 resembles points made by contemporary proponents of more generous welfare benefits (e.g. [5]). All six pairs of issues had an easily identifiable ‘right’ and ‘left’ point of view, but it was not assumed that everyone holds homogeneously ‘rightwing’ or ‘leftwing’ attitudes towards all six issues. Indeed, as we shall see shortly, very many respondents hold some of each.

After making their newsworthiness ratings, participants were asked to provide an explicit opinion on the six issues around which the items were constructed, as well as answering a few more general questions about political ideology and political interest.

## 2. Method

### 2.1. Participants

Participants were recruited from Amazon Mechanical Turk. To be eligible, the subjects had to be US residents who had completed at least one prior experiment with ‘approved’ performance in at least 95% of their prior jobs. A total of 569 subjects completed the study in return for payment. Subjects were randomly assigned to one of the four between-subject conditions. When the first 260 subjects had completed the study, those data were downloaded from the server and analysed, with results reported below. The sample consisted of 131 men and 129 women. The mean age was 34.8 years with a standard deviation of 12.4. The oldest individual in the sample was 68 years old.

Meanwhile, accrual of additional data continued, providing an additional 309 subjects. This latter dataset was retained separately on the server as a hold-out replication sample, and examined only after all of the statistical analysis described below had been computed.

### 2.2. Materials

A list of 12 hypothetical news stories was constructed (see appendix A). The 12 stories consisted of six pairs of stories relating (directly or indirectly) to the topics of welfare, affirmative action, military spending, police racial bias, support for gay rights and environment (species conservation). Pairs were constructed so that one news development seemed to provide obviously useful ‘ammunition’ for one side of a particular issue, while the other item in the pair provided ammunition for the other side. These are referred to as different ‘versions’ of the issue question. An additional eight filler items were included.

### 2.3. Procedure

The data collection was approved by the University of California San Diego Institutional Review Board. Participants provided informed consent by clicking their assent to the informed consent participation form. Next, they completed nine demographic questions (no political questions were asked at this phase).

After that, the subjects performed the newsworthiness rating task. The instructions preceding this task are provided in full in appendix D. The instructions were constructed to emphasize strongly that subjects should judge the *importance* of each story, and, more specifically, that they should assess the importance that the story would have for the *readership of a city newspaper*. It was pointed out that, in this context, national stories should not necessarily be rated as having greater newsworthiness than local stories. To ensure that all subjects understood their task, a single-item quiz was provided before the experiment began. Subjects were asked the following question:

*Just to be sure you understand the task, please indicate what you will be rating:*

1. How interesting I personally find a story to be.
2. How important I would judge the story as being if I were the editor of a general-circulation newspaper.
3. How long the story is.

The correct answer was choice 2. If subjects made any other choice, they were required to reread the instructions and then to take the quiz again until they selected the correct answer.

The 12 critical and eight filler items were then presented in a random order, individually selected for each subject.

After the newsworthiness rating task, the subject rated their general, social and economic political attitudes on a scale from 1 (very conservative) to 7 (very liberal). Next, they indicated their opinion on each of the six issues that underlay the 12 news stories they had rated in the first phase of the study. Finally, subjects were asked about their degree of interest in news and in politics.

## 3. Results

### 3.1. Political views of sample

**Figure 1.**
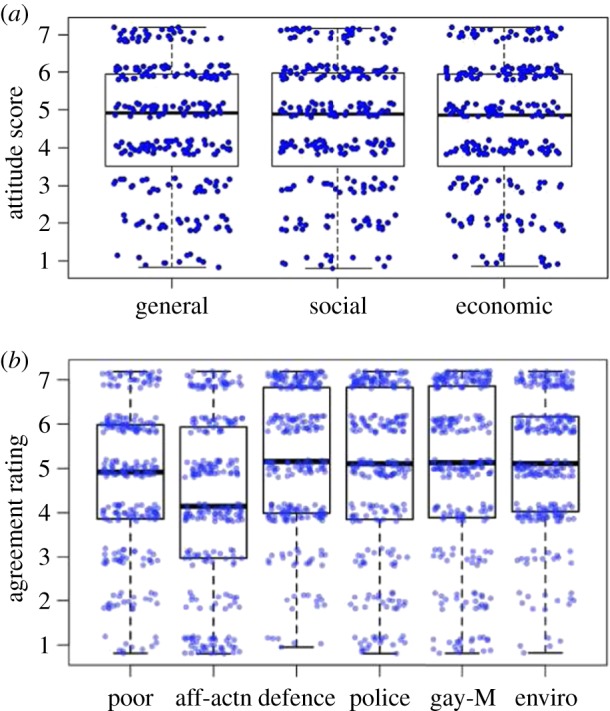
Distribution of the sample's views on the general political attitude questions (*a*) and each of the six political issues covered in the current study (*b*). (Exact questions are all listed in appendix B and ‘poor’ is reverse-scored). The thick black line shows the mean, while the thinner lines that constitute each box show the 25th and 75th percentiles, with the raw datapoints shown in blue (random horizontal and vertical jitter was added for display purposes). The figure shows that the sample clearly leans towards a liberal view, except for affirmative action, where all views receive considerable support.

Figure 1 shows boxplots of the distribution of the sample's opinions on the general attitude measures (figure 1*a*) and on all six issues (figure 1*b*; for this calculation, one issue was reverse-scored: issue 1, Aid to Poor).

Plainly, the sample shows predominantly liberal views on most issues (affirmative action being an exception). This fits with prior reports of the characteristics of Mechanical Turk samples [6].

Table 1 presents the intercorrelation matrix of the six issue questions seen in figure 1, plus the three additional questions asking subjects to characterize their overall political ideology. The three general political orientation descriptors (general, economic and social conservatism) are, not surprisingly, fairly strongly correlated with each other (in the range from 0.6 to 0.9). The specific issue questions (affirmative action, environment, etc.) are correlated with each other to a more moderate degree, typically about 0.4–0.5). This fits with previous observations that many people in the general public hold issue views that would not be viewed as consistent for those who habitually view all issues from a right–left perspective [7].

**Table 1.** Intercorrelation matrix for all the political questions in the survey. The first three measures are general measures of liberalism, and, as expected, these are highly intercorrelated. Measures os1–os6 are issue attitude questions associated with the six news topics. These issue questions show more moderate inter-correlations.

	general_polit	social_polit	econ_polit	os1	os2	os3	os4	os5	os6
general_polit									
social_polit	0.817								
econ_polit	0.742	0.640							
os1	−0.570	−0.560	−0.584						
os2	0.334	0.298	0.372	−0.410					
os3	0.404	0.383	0.371	−0.353	0.329				
os4	0.485	0.456	0.460	−0.527	0.546	0.386			
os5	0.496	0.457	0.461	−0.528	0.547	0.388	0.963		
os6	0.471	0.435	0.393	−0.343	0.456	0.411	0.470	0.462	

### 3.2. Reliability of newsworthiness judgements

To determine whether newsworthiness judgements have psychometric coherence, Cronbach's alpha was computed for the eight filler items (appendix C) using the R package Psy. The value was 0.61 (95% CI = 0.52, 0.7), suggesting moderate coherence. For the critical items, Cronbach's alpha was actually slightly higher: 0.71. These values would normally be considered ‘acceptable’ for measurements of a unidimensional scale. On the other hand, the partisan bias hypothesized in the current study would be expected to attenuate Cronbach's alpha, as would sampling noise and possibly other unknown bias factors. Thus, the fact that these values are well short of 1.0 is not discouraging for the hypothesis being examined here.

### 3.3. Do issue views bias newsworthiness judgements?

The key hypothesis motivating the present study was that people's views on a given issue will predict differences in their ratings of the two hypothetical news stories associated with that issue. The difference was expected to reflect a bias towards rating as more newsworthy any story that provides partisan ammunition for the rater's preferred issue position. For each of the six issues, the hypothesis examined here predicted a specific form of interaction. All the predicted interactions were present in the average results at least to some degree, with four of the six showing a clearcut crossover interaction. We first describe the interactions qualitatively and graphically, and then describe statistical tests for the interactions.

For each of the six issue questions, we compared subjects who provided a rating less than 4 (*low* raters for that question) and those with a rating greater than four (*high* raters for that question). Subjects providing a neutral rating (choice 4 on a 1–7 scale) were not included in this analysis. Table 2 shows the number of subjects rating low and high on each of the six issue questions.

**Table 2.** Number of subjects holding the low and high opinion on each issue.

	issue 1	issue 2	issue 3	issue 4	issue 5	issue 6
low	59	124	169	170	172	176
high	149	81	34	59	59	29

**Figure 2.**
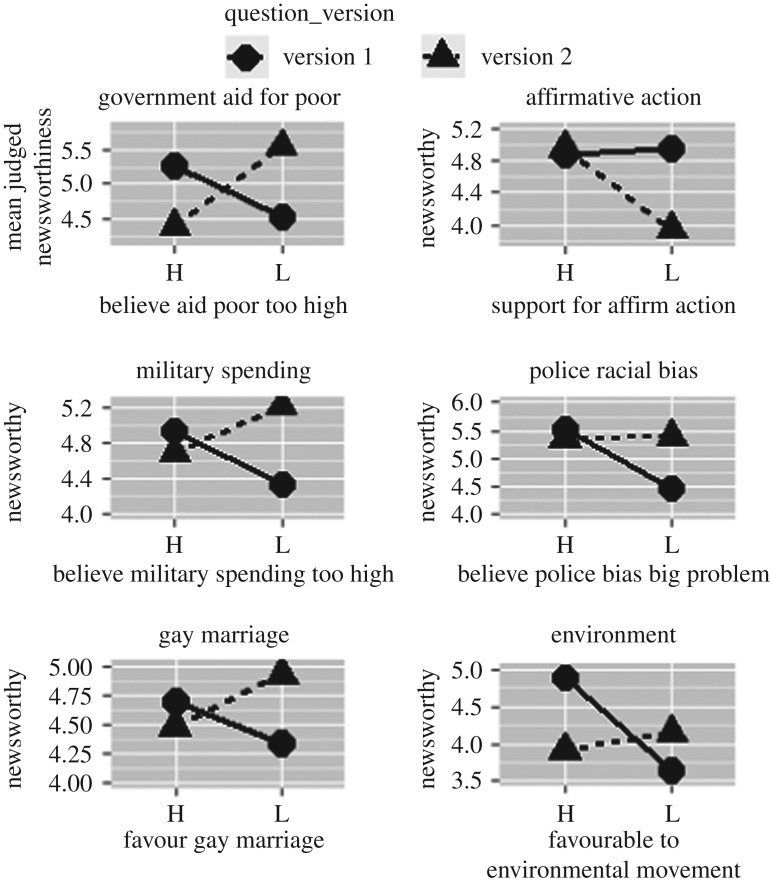
Mean newsworthiness rating as a function of the subjects' position on issue (high versus low) and which version of the news story was being rated.

Figure 2 shows the average newsworthiness rating for the two scenarios related to each of the six issues, with a separate panel for each issue. On the first issue, government aid for the poor, people who felt government aid for the poor in the USA was too high rated version 1 (a story about welfare fraud) as greater in newsworthiness than version 2 (a story about a study showing that food stamp recipients were not receiving enough aid to allow them to prepare nutritious meals). Subjects who did not agree that aid for the poor was too high ranked the two stories in the opposite fashion.

For issue 2, affirmative action, those who opposed affirmative action rated a news story about wealthy recipients of affirmative action benefits (version 1) as much more important than a news story about declines in minority enrolment resulting from elimination of affirmative action in certain states (version 2), while the opposite was true (to a slight extent) for those supporting affirmative action.

On issue 3, military spending, those who believed US military spending was too high rated as more newsworthy a story about the enormity of US military expenditures (version 1) compared with those of other countries than a story suggesting that US defence capabilities had diminished below some potentially important level (version 2). For those who rejected the claim that US military spending is too low, this newsworthiness ranking was inverted.

On the topic of police bias, those viewing racial bias in police as a very serious problem rated as highly newsworthy a story about citizen expressions of upset about police tactics in the city (version 1) as slightly more newsworthy than a story about increasing robbery rates in the downtown area of the city (version 2). Those who did not view racial bias in the police as a very serious problem rated the former much less important than the latter.

On the topic of gay marriage, subjects were asked to evaluate the newsworthiness of two news stories involving a hypothetical Eagle Scout. Those in favour of gay marriage found the scenario ‘A local Eagle Scout was arrested for a hate crime’ (version 1) more newsworthy than ‘A local Eagle Scout saved a woman from a mugger’ (version 2). Those opposing gay marriage ranked the two in the opposite fashion.

Finally, on the topic of the environment, subjects who described themselves as supporters of the environmental movement rated as more newsworthy a story about a study showing that endangered species were in greater danger than previously thought (version 1) than a study showing the opposite (version 2). The ranking was reversed for those who would not characterize themselves as supporters of the environmental movement.

### 3.4. Statistical tests of interactions

Each of the six interactions discussed above was assessed with a separate analysis of variance, with newsworthiness rating as the dependent variable, and two independent variables: issue attitude (high versus low) and question version. Table 3 shows the *F* values and significance levels for the issue belief × question version interaction terms. All predicted interactions were significant.

**Table 3.** Interactions between question attitude and question version. For all six topics, results confirm the predicted interaction. *η^2^* indicates effect size (proportion of variance accounted for by the interaction).

issue	*F*	*p*	d.f.	MSe	*η^2^*
aid for poor	38.8	0.001	1, 206	73.5	0.067
affirmative action	14.0	0.001	1, 203	29.7	0.028
police bias	12.5	0.001	1, 201	17.7	0.017
military spending	19.3	0.001	1, 227	25.9	0.026
gay marriage	8.5	0.005	1, 229	14.4	0.011
environment	21.6	0.001	1, 203	26.3	0.027

### 3.5. Overall left–right newsworthiness bias

So far we have examined each issue (and the question related to that issue) separately from the other issues. However, as shown in figure 2, there is a substantial (although, as noted above, far from perfect) correlation between attitudes to each of these issues and political ideology assessed on the commonplace left–right axis. A broad measure of each subject's left-versus-right newsworthiness bias was constructed by adding up all of the subject's newsworthiness scores for the version of each question that provides ‘ammunition’ for the conservative point of view (items 1, 3, 6, 8, 10 and 12), and subtracting all of the subject's importance ratings for the versions reinforcing liberal points of view (items 2, 4, 5, 7, 9 and 11).

**Figure 3.**
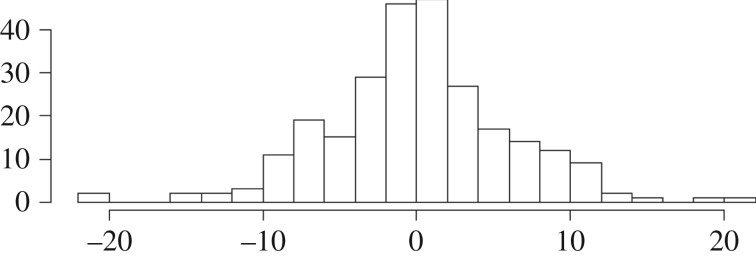
Distribution of left–right newsworthiness bias aggregated over items.

This measure (*L-R newsworthiness bias*) has a roughly Gaussian distribution (figure 3). In our dataset, it was quite strongly correlated with the subject's self-reported overall political orientation *r* = 0.337, *p* = 0.00000001, CI = (−0.44, −0.22).

Overall political ideology is substantially correlated with the tendency to judge as more newsworthy stories providing ‘ammunition’ for the subject's overall political affiliation.

### 3.6. Individual differences in newsworthiness bias

**Figure 4.**
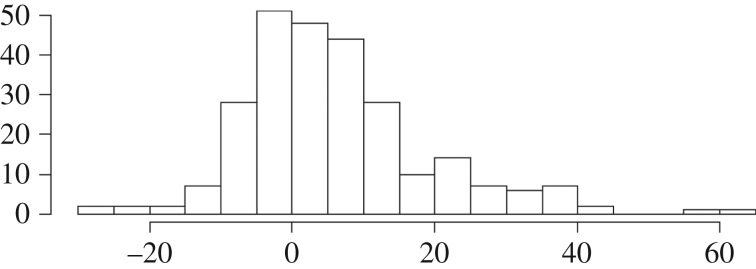
Distribution over subjects of newsworthiness bias (tendency, aggregated across the six issues, to rate as more newsworthy any news story providing ammunition for the subject's own view of the issue).

It is also of interest to measure individual differences in people's tendency to conflate newsworthiness (which we defined to be the importance that the story would have for the *readership of a city newspaper*) with political usefulness. To do so, we computed the following index:

$$\sum_{\mathrm{Issue}\;i}\text{(opinion on issue}\;i)\times (N(\mathrm{Vers}\;A)-N(\mathrm{Vers}\;B)),$$

where *N*(Vers *x*) is the subject's rating of newsworthiness of version *x* of the question related to the issue (where version A is whichever version tends to back up the opinion measured in the opinion question and version B tends to oppose it). Thus, this measure basically answers the question ‘to what degree does this subject tend to judge as more newsworthy stories which provide support for their own view of an issue, whatever that might be?’. Figure 4 shows the distribution of this measure in the sample.

Bias score was very weakly but significantly correlated with general political liberalism, *r* = 0.129, *p* < 0.04, and with interest in politics, *r* = 0.136, *p* < 0.03. It was not significantly correlated with self-reported tendency to follow the news closely (*r* = 0.086). The correlation with interest is not surprising: interest in politics is known to be related to holding strong opinions.

### 3.7. Hold-out replication sample

As mentioned above, a hold-out replication sample was retained to provide a rough indicator of the robustness of the findings reported. This sample (*n* = 309) was retained on the server in an unexamined state while all of the statistics reported above were computed. After completion of the analyses reported above, on 12 June 2015 the hold-out data were downloaded and analysed with the same R script. Within the hold-out sample, the direction of all of the interaction terms was in the same (predicted) direction, although in the hold-out sample some of the interactions were not significant. The overall bias measure (correlation of political conservatism with left–right bias on the newsworthiness judgements) was significant in the hold-out sample although slightly smaller in magnitude (0.23 in the hold-out versus 0.34 in the initial sample, both significant). On the other hand, the correlation of political liberalism with overall (non-directional) issue bias was again significant but larger in magnitude (0.27 versus 0.13 in the original sample). In general, the hold-out sample provided a very satisfactory direct replication of the conclusions drawn here.

## 4. General discussion

The results clearly show that judgements of newsworthiness—specified as importance to a hypothetical newspaper readership—are contaminated by an ideological bias: news stories that offer good ‘ammunition’ for the views of the rater are assigned a higher news value than those providing ammunition for the opposing view. The bias appeared slightly greater for those reporting an interest in politics and—for unknown reasons—slightly greater for those characterizing their political views as liberal. The results are not easily interpreted in terms of a simple uncertainty reduction account of news value. If interest and newsworthiness are enhanced by surprise (as they surely are in general according to both common sense and much prior research; see [3]), then ideological differences should, if anything, have gone in the other direction. Consider, for example, the gay marriage case. If opponents of gay marriage hold a more cynical view of Boy Scouts based on resentment of the organization's positions with respect to gay rights, then they ought to find the arrest of a Boy Scout *less* surprising than would a gay marriage proponent—not more surprising. Similar arguments would seem to apply to several of the pairs of hypothetical news stories. What evidently receives higher ratings of news value are not stories that are surprising to the rater—but rather stories that the rater feels other people *ought to pay attention to*—items thought likely to compel a neutral party to reach the same opinions as those held by the rater.

### 4.1. Rationality and biases

The bias shown here is broadly congenial to much evidence in the psychological literature showing widespread partisan biases in handling and appraisal of information. One example is the phenomenon of *biased assimilation,* whereby people evaluate arguments whose outcomes favour their own positions as being more credible (see [8], for discussion). Many writers have pointed out that biased assimilation is not necessarily irrational. Given a strong basis for believing conclusion X, presuming that evidence tending to rebut X is less credible is in line with Bayesian precepts [9]. The present bias may be harder to defend as rational. After all, subjects in the present study were told to rate based on how interesting it would be for potential readers of a city newspaper, not how useful to the rater's own political faction it would be for the story to draw a large readership.

The phenomenon discussed here seems broadly congenial to the idea proposed by some evolutionary and political psychologists that people have a strong but unwitting tendency to reach moral and factual judgements consonant with their self-interest and/or their desire to advance their own political faction or ‘team’ (e.g. [10–12]).

The results are also obviously consistent with observations that media outlets make greater use of press bulletins released by political factions that they tend to support in editorial matters [13,14]. The current results also complement ‘selective exposure theory’ [15,16], which states that people tend to seek out information that supports their own opinions. The current results suggest that they naturally seek out the same sort of information for others to read.

### 4.2. Limitations and future directions

The results provide clear support for the idea that, when people try to assess the newsworthiness of hypothetical news stories, their judgements are biased by the degree to which the news story provides ‘ammunition’ for their own political beliefs and preferences: the better the ammunition, the greater their estimates of the news value of a story. This hypothesis generated six predictions of interactions and all six were confirmed, with many distinctive crossover interactions (and all were directionally confirmed within a hold-out replication sample). Nonetheless, there are many limitations of the present work to which we would draw the reader's attention (each of these caveats points to potentially interesting directions for follow-up research).

We have described the phenomenon being examined here as a bias to rate as newsworthy stories *likely to persuade other people to share the participants’ own political values*. Could one argue that the bias does not reflect a desire to accumulate political ‘ammunition’? Rather, might people who believe that problem X is a serious problem (and thus favour strenuous efforts being made to solve the problem) simply wish for others to be made more *aware* of problem X? While the two interpretations are conceptually very similar and even close to identical in their predictions with regards to biases in media story selection, they do seem potentially logically distinguishable.

It seems to us that, while most of the scenarios examined here could be interpreted in either way, at least one set of items—the questions relating to the gay marriage item—do not lend themselves quite so well to the awareness interpretation. The Boys Scouts of America organization was widely perceived as opposing gay rights. A story about an individual Eagle Scout engaging in the good behaviour, or hateful behaviour, is likely to symbolically validate the attitudes of gay marriage opponents, and proponents, respectively. It seems far-fetched to suppose that anyone could view such a story as having great ‘social significance’ in the sense described by Shoemaker & Cohen [3]. In follow-up work, it would be quite interesting to explore additional items of this kind, where the news story has ‘ammunition value’ but little broad social significance.

One clear limitation of the current study is that the subjects were drawn from a convenience sample of US residents, not a sample of professional journalists or people with journalism training. It certainly cannot be ruled out that training in a journalism school and/or experience working in an editorial position might permit people to transcend the bias revealed here, with skilled practitioners making purer and more refined newsworthiness judgements. On the other hand, it seems a bit hard to believe that this bias would be eradicated in journalistic education if, as appears, it is scarcely discussed in writings that would be read by journalism students. After all, debiasing is normally assumed to require explicitly labelling and noting of a potential bias (see [17]). An investigation of this question within a sample of trained journalists would seem to be in order.

Another limitation of the current study is that we did not specifically demonstrate that the news stories examined here actually have the persuasive effect that we have assumed people believe them to have. It would be interesting to do an experiment to verify that they do. This would be challenging, however, because inducing shifts in opinions would presumably require making people believe that the stories are actually true rather than hypothetical.

## 5. Concluding remarks

The bias described here seems interesting not only because it is congenial to the emerging view that people commonly engage in a continuous process of motivated reasoning or ‘spin-doctoring’ in constructing many kinds of political and moral judgements (see [7,11]), but also because it may have practical significance with regard to journalistic practice. Standard norms of ‘reputable’ journalists include taking precautions against biased reporting of specific stories, e.g. seeking comment from spokesmen for both sides of any ongoing dispute. Those formulaic rules of journalism, while important, do not provide any effective resistance to biases that arise earlier, in the process of deciding which stories are newsworthy enough to deserve coverage in the first place. The current results suggest that biases in this phase might be strong and insidious.

## Ethics

The research was approved by the Social Sciences Institutional Review Board of the University of California, San Diego (Project no. 101193XX).

## Data accessibility

The data and materials are available at https://figshare.com/articles/Materials_for_Pashler_Heriot_Perceptions_of_Newsworthiness_are_Contaminated_by_a_Political_Usefulness_Bias_/4307813.

## Authors' contributions

H.P. and G.H. conceived of and designed the study and study materials, planned the analyses, interpreted the results and wrote the manuscript. H.P. supervised data collection and conducted the analyses.

## Competing interests

The authors declare no competing interests.

## Funding

The work was supported by funds provided by the University of California, San Diego.

## Appendix A. Issue-related hypothetical news stories

1. A welfare recipient in your city was arrested for fraudulently receiving $25 000 worth of welfare cheques over a 2 year period.
2. A new study shows that food stamp recipients in your state do not receive enough food stamps to cover the cost of preparing nutritious meals for their households (as defined by the US Government Department of Health and Human Services).
3. A new study shows that almost half of the construction contractors receiving affirmative action benefits in your state have personal assets in excess of 1 million dollars.
4. A new study shows that, in states that have banned affirmative action, the number of minority students in these states' university systems has fallen by 25%.
5. A study showing that the USA spends more on its military than the next eight countries combined.
6. A study finding that the US military probably lacks the capability of fighting two wars at the same time, a goal long specified in US military doctrine.
7. A story about a hearing held by the police board in your city at which citizens expressed concern that police tactics used in the African–American community are counterproductive.
8. A story reporting an 18% increase in robberies over the last 5 years in the downtown area of your city.
9. A local Eagle Scout was arrested for a hate crime.
10. A local Eagle Scout saved a woman from a mugger.
11. A new study finds many endangered species actually closer to extinction than previously believed.
12. A new study concludes that many endangered species are actually more plentiful than previously assumed.

## Appendix B. Issue attitude questions

OS1. Please indicate your views of present levels of government aid for the poor:
  1 = much too low; 4 = about right; 7 = much too high
OS2. What is your general attitude to affirmative action for women and minorities?
  1 = strongly oppose; 7 = strongly favour
OS3. Please indicate your views on present levels of US government defence spending:
  1 = much too low; 4 = about right; 7 = much too high
OS4. In your opinion, is racial bias in police departments a big problem?
  1 = not a very big problem; 7 = extremely big problem
OS5. What is your opinion about gay marriage?
  1 = strongly opposed; 7 = strongly supportive
OS6. What is your attitude to the environmental movement?
  1 = strongly disapprove; 7 = strongly approve

## Appendix C. Filler news stories

F1. A story reporting that the mayor of another major city in your state (not your city) was arrested for bribery.
F2. A story reporting that a man in your city was arrested for deliberately running his car into a neighbour's vehicle due to aggravation over the neighbour's dog barking.
F3. A story reporting that a prominent movie actress habitually used cocaine while a college student.
F4. A story reporting that a prominent movie actress purchased marijuana at a legally authorized outlet while visiting Colorado last week.
F5. A story reporting that the sections of Interstate 1 that run through your city will be undergoing $20 million worth of repairs over the next six months and will probably result in minor traffic delays over that period.
F6. A story on a six-car pile-up on Interstate 1 on the south side of your city, resulting in serious injuries to three people, but no deaths.
F7. A story reporting toy industry analysts' speculations about what the hot new toy will be at Christmas this year.
F8. A story describing President Obama's extensive collection of baseball cards, which he has been collecting because his grandfather introduced him to the hobby 45 years ago.

## Appendix D. Instructions on newsworthiness rating task

For the next 12 questions, we want you to imagine that you are an editor of the major general-circulation newspaper in a medium-sized city. Editors have to decide on the importance of news stories in order to determine how prominently to place them in the newspaper. For example, a very important story might go on page 1 while a relatively unimportant story might go elsewhere in the paper. We will show you a series of hypothetical news stories. For each one, we would like you to estimate its importance. The scale ranges from 1 = very unimportant; place in low-priority spot in paper to 7 = extremely important; place in prominent position on page 1.

Note that when an item refers to ‘your city’ or ‘your state’, that means the city and state that the newspaper you are editing is located in.

Again, what you will be rating is just *importance to your readership*, not necessarily what page it will go on. Do not assume that national stories are necessarily more important or deserve more prominence—general-circulation newspapers often have a mix of local and national stories both on the front page and in the back pages.
